# Vancomycin-resistant Gram-positive bacterial endophthalmitis: epidemiology, treatment options, and outcomes

**DOI:** 10.1186/1869-5760-3-46

**Published:** 2013-04-22

**Authors:** Manav Khera, Avinash Pathengay, Animesh Jindal, Subhadra Jalali, Annie Mathai, Rajeev Reddy Pappuru, Nidhi Relhan, Taraprasad Das, Savitri Sharma, Harry W Flynn

**Affiliations:** 1 LV Prasad Eye Institute, GMR Varalakshmi Campus, Visakhapatnam 530040, India; 2 Srimati Kanuri Santhamma Centre for Vitreoretinal Diseases, LVPEI, KAR Campus, Hyderabad 500034, India; 3 LVPEI, Bhubaneswar Campus, Bhubaneswar, Odisha 751024, India; 4 Bascom Palmer Eye Institute, Department of Ophthalmology, University of Miami Miller School of Medicine, Miami, FL 33136, USA

**Keywords:** Antibiotics, Antibiotic resistance, Endophthalmitis, Gram-positive organisms, Vancomycin, Vitrectomy

## Abstract

**Background:**

The purpose of this study is to evaluate the microbiological profile and treatment outcomes of vancomycin-resistant Gram-positive bacterial endophthalmitis. Medical records of all patients with Gram-positive bacterial endophthalmitis resistant to vancomycin presenting between 1 January 2005 and 31 December 2010 were reviewed in this noncomparative, consecutive, retrospective case series. Favorable outcome was defined as a best-corrected visual acuity of ≥20/200.

**Results:**

Out of 682 culture-positive endophthalmitis isolates, 448/682 (65.6%) were associated with Gram-positive bacteria. *In vitro* resistance to vancomycin was noted in 7/448 (1.56%). Three cases were posttraumatic, three were postoperative, and one was endogenous in origin. Four *Bacillus* isolates, two *Staphylococcus* isolates, and an *Enterococcus* isolate were resistant. Isolates resistant to vancomycin were sensitive *in vitro* to ciprofloxacin in 6/7 (86%) patients. Presenting visual acuity was light perception in all seven cases. Favorable outcome was achieved in only 1/7 (14.3%) cases.

**Conclusions:**

Vancomycin-resistant endophthalmitis is uncommon and usually associated with poor visual outcome. *Bacillus* sp. is the most frequent Gram-positive bacteria resistant to vancomycin. Fluoroquinolones like ciprofloxacin may be considered as a useful alternative in vancomycin-resistant endophthalmitis.

## Background

Management of endophthalmitis includes performing either vitreous tap/biopsy or vitrectomy and institution of appropriate intravitreal antibiotic therapy. Gram-positive bacteria are the most common cause of both exogenous and endogenous endophthalmitis. Vancomycin is consi dered to be the drug of choice for empiric coverage of Gram-positive organisms in endophthalmitis. However, there are few reports of vancomycin resistance in Gram-positive bacterial isolates (*Enterococcus* sp. and *Staphylococcus* sp.) associated with endophthalmitis [1,2]. The current study evaluates microbiological characteristics and visual outcome of vancomycin-resistant Gram-positive bacterial endophthalmitis in a large clinical series.

## Methods

In this noncomparative, consecutive case series, the medical records of all patients with culture-proven, clinically diagnosed, infective bacterial endophthalmitis between January 2005 and December 2010 were reviewed after obtaining institutional review board approval. The study followed the Declaration of Helsinki guidelines. Data collected included demographic details, etiology, complications of surgery, duration of symptoms, interval between the event and presentation to the institute, use of medication (topical or systemic), examination findings (presenting visual acuity, presence of hypopyon, presence of vitreous cells, and optic disk visibility), ultrasonography (with attention to the presence of vitreous membranes), and microbiology (including culture and sensitivity). The Endophthalmitis Vitrectomy Study recommendations [3] (vitrectomy reserved for eyes with presenting vision of light perception and vitreous biopsy for eyes with presenting vision of hand motions at 1 m or more) were followed in postoperative endophthalmitis eyes only. Vitrectomy was performed in all eyes with traumatic and endogenous endophthalmitis. All eyes received intravitreal vancomycin (1.0 mg in 0.1 ml) and amikacin (0.4 mg in 0.1 ml) or ceftazidime (2.25 mg in 0.1 ml). Treatment and management decisions were made by the individual treating physicians without a predefined study protocol. The undiluted vitreous biopsy samples were stained with Gram stain for microscopy and also inoculated directly onto 5% sheep blood agar, chocolate agar, Sabouraud dextrose agar, thioglycollate, and brain-heart infusion broth. A culture was considered positive when there was growth of the same organism on two or more media, confluent growth at the site of inoculation on one solid medium, or growth in one medium with consistent microscopy findings. Antibiotic sensitivity was tested by disk diffusion technique. A best-corrected visual acuity of 20/200 or more at final follow-up was defined as a favorable outcome.

## Results

Of 682 culture-positive endophthalmitis, 448 (65.6%) were associated with Gram-positive bacteria. Resistance to vancomycin was noted in 7 (1.56%) cases. Mean age of the patients was 35 years (range 4 to 70 years). Three cases were posttraumatic, three were postoperative, and one was endogenous in origin. All cases, except the one with endogenous endophthalmitis, presented within 1 week of symptoms. Presenting visual acuity was only light perception in all seven cases. The details of the primary and secondary interventions are summarized in Table 1. Vancomycin resistance was commonly noted in *Bacillus* sp. (4 of 96, 4.17%), followed by *Staphylococcus* sp. (2 of 80, 2.5%) and *Enterococcus* sp. (1 of 5, 20%). Six of the seven isolates also had multidrug resistance. Six (86%) isolates were sensitive to ciprofloxacin followed by 4 (57%) each to gentamicin, gatifloxacin, and ceftazidime (Table 2). Favorable outcome was achieved in 1/7 (14.3%) patients. Five eyes went into phthisis, while one patient achieved counting fingers at 1 m.

**Table 1:** Demographic characteristics, etiology, management, and outcomes of patients with vancomycin-resistant endophthalmitis due to Gram-positive bacteria

**Serial number**	**Age (years)**	**Sex**	**Etiology**	**Duration (days)**	**Presenting V/A**	**Primary intervention**	**Secondary intervention**	**Smear**	**Systemic antibiotic**	**Intravitreal antibiotic**	**Intravitreal steroid**	**Final V/A**	**Follow-up (months)**	**Outcome**
1	20	M	Trauma	1	LP	CTR + PPV + IOAB	PPV + M + C + T	GPB	Ciprofloxacin	V + C	T	CF - 1 m	3	Unfavorable
2	70	F	Postcataract surgery	5	LP	PPV + IOAB + IOL explant	Nil	GPC	Gatifloxacin	V + A	D	LP	3	Unfavorable
3	70	M	Postcataract surgery	1	LP	PPV + IOAB	C + T	GPB	Ciprofloxacin	V + A	T	20/20p	4	Favorable
4	5	M	Trauma	1	LP	CTR + PPL + PPV + IOAB	Nil	GPB	Cephalexin	V + C + Am	-	LP	6	Unfavorable
5	4	F	Trauma	3	HM	CTR + PPL + PPV + IOAB	Nil	GPB	Ciprofloxacin	V + C	-	LP	3	Unfavorable
6	60	M	Postcataract surgery	6	LP	PPV + IOAB	Nil	GPC	Ciprofloxacin	V + C	-	LP	6	Unfavorable
7	16	F	Endogenous	20	LP	PPV + IOAB	Enucleation	GPC	Ciprofloxacin	V + A	-	NLP	6	Unfavorable

C, ceftazidime; T, triamcinolone; D, dexamethasone; V, vancomycin; A, amikacin; M, membranectomy; GPC, Gram-positive cocci; GPB, Gram-positive bacilli; CTR, corneal tear repair; PPV, pars plana vitrectomy; IOAB, intraocular antibiotic; PPL, pars plana lensectomy; Am, amphotericin B; CF, counting fingers; LP, light perception; NLP, no light perception; V/A, visual acuity.

**Table 2:** Antimicrobial susceptibility of vancomycin-resistant Gram-positive organisms

**Number**	**Bacteria**	**Cefa**	**Cefta**	**Ch**	**Gen**	**Cipro**	**Gati**	**Oflox**	**Vanco**	**Amika**
1	*Bacillus macerans*	R	S	R	S	S	R	R	R	S
2	*Staphylococcus epidermidis*	R	R	R	R	S	S	S	R	S
3	*Bacillus* species	R	S	R	S	S	S	S	R	S
4	*Bacillus cereus*	R	R	R	S	S	R	R	I	R
5	*Bacillus* species	S	S	S	S	S	S	S	R	S
6	*Enterococcus faecalis*	R	R	R	R	R	R	R	I	R
7	*Staphylococcus aureus*	S	S	R	R	S	S	R	I	R

Amika, amikacin; Cefa, cefazolin; Cefta, ceftazidime; Ch, chloramphenicol; Gen, gentamicin; Cipro, ciprofloxacin; Gati, gatifloxacin; Oflox, ofloxacin; Vanco, vancomycin; S, sensitive; R, resistant; I, intermediate.

## Discussion

Vancomycin is a widely used glycopeptide antibiotic that is effective against most Gram-positive bacteria including *Streptococcus*, *Staphylococcus*, and *Bacillus* species. It is commonly used for the treatment of methicillin-resistant coagulase-negative *Staphylococcus* and methicillin-resistant *Staphylococcus aureus* (MRSA) infections. Vancomycin kills bacteria by binding irreversibly to d-alanyl-d-alanine moieties of the *N*-acetylmuramic acid (NAM) and *N*-acetylglucosamine (NAG) peptides. This inhibits the synthesis and cross-linking of the NAM/NAG polymers that form the backbone of the cell wall. Resistant mutants are very rare, except for vancomycin-resistant *Enterococcus*. The mechanism of acquiring vancomycin resistance includes conversion of d-Ala-d-Ala to the depsipeptide d-Ala-d-Lac or to d-Ala-d-Ser, leading to altered cross-linkages in the peptidoglycans of the cell wall [4].

In the Endophthalmitis Vitrectomy Study, 100% of the Gram-positive bacteria were susceptible to vancomycin [5]. However, in the current series, three acute-onset postoperative endophthalmitis patients with Gram-positive organisms (*Staphylococcus epidermidis*, *Bacillus* sp., and *Enterococcus faecalis*) were resistant to vancomycin. There have been reports of using vancomycin as a prophylaxis either intracamerally or in the irrigating solution during cataract surgery [6,7] which may possibly increase the emergence of vancomycin-resistant Gram-positive bacteria. In the present series, *Bacillus* sp. was the most common Gram-positive bacteria resistant to vancomycin. In a previous report, resistance among *Bacillus* sp. to vancomycin was noted in 10 of 31 (32.3%) organisms isolated between 1991 and 1998 [8]. In another large case series reported by Miller and associates, all of the *Bacillus* sp. isolates were sensitive to vancomycin [9].

In the current study, most of the patients had poor visual outcomes. This can be attributed to multiple factors including initial delay in presentation, microbial resistance, and virulence of the organism, as well as concurrent trauma in some patients. It was interesting to note that these vancomycin-resistant Gram-positive organisms were most likely to be sensitive to the second-generation fluoroquinolone ciprofloxacin.

Quinupristin-dalfopristin, linezolid, daptomycin, and tigecycline have been used to treat systemic infections caused by vancomycin-resistant bacteria, though there are no formal recommendations for treatment of such endophthalmitis cases, given the paucity of these infections. Quinupristin/dalfopristin is a synthetic, parenteral, streptogramin antibiotic that acts by sequential ribosomal binding to inhibit protein synthesis and is effective *in vitro* against *Enterococcus faecium*, MRSA, *Streptococcus pneumoniae*, and other Gram-positive cocci. Hernandez-Da Mota reported successful treatment of endophthalmitis due to vancomycin-resistant *S. aureus* by administration of intravitreal 0.4 mg/0.1 ml quinupristin/dalfopristin injection [10]. Linezolid is an oxazolidinone antibiotic that inhibits protein synthesis by binding to domain V of the 235 ribosomal RNA of the 50S subunit of bacterial ribosomes and is active *in vitro* against vancomycin-resistant *Enterococcus*, MRSA, vancomycin-resistant *S. aureus*, and penicillin-resistant *S. pneumoniae*. Systemic linezolid has been reported in the successful treatment of vancomycin-resistant *E. faecium* endophthalmitis following penetrating keratoplasty [11]. Daptomycin is a lipopeptide that binds to the bacterial cytoplasmic membrane to effect release of intracellular ions and cell death. It demonstrates good *in vitro* bactericidal activity for vancomycin-resistant strains of bacteria accounting for endophthalmitis, including *S. epidermidis*, *S. aureus*, *S. pneumoniae*, *E. faecalis*, and *E. faecium*[12]. Comer and colleagues demonstrated that administration of 200 μg of intravitreal daptomycin was safe and efficacious in a rabbit model of bacterial endophthalmitis [13]. Tigecycline is a glycylcycline antibiotic that binds to the 30S ribosomal subunit to inhibit protein synthesis. It is active *in vitro* against MRSA, other multidrug-resistant Gram-positive cocci, and many Gram-negative bacilli [14].

Limitations of this report include the fact that the isolates were vancomycin-resistant by disk diffusion method which was not confirmed by MIC and the small number of cases in this retrospective series. However, it should be noted that these vancomycin-resistant organisms have been reported to be resistant to multiple other antibiotics [15]. In the current series, six of the seven isolates showed multidrug resistance.

## Conclusions

In conclusion, vancomycin resistance among Gram-positive isolates is an uncommon but an emerging problem. The magnitude of antibiotic resistance requires ongoing surveillance and periodic reporting from individual laboratories.

## Competing interests

The authors declare that they have no competing interests.

## Authors’ contributions

MK and AJ carried out the data collection and data analysis and drafted the manuscript. AP is one of the treating physicians and also carried out the correction of the manuscript. SJ, AM, RRP, NR, and TD are the other treating physicians. SS is the microbiologist. HWFJ corrected the manuscript. All authors read and approved the final manuscript.
